# Selective targeting of *Plasmodium falciparum* hexose transporter by phytochemical Ginsenoside Rg1 disrupts glucose metabolism and blocks development of parasite

**DOI:** 10.1128/aac.01727-25

**Published:** 2026-06-12

**Authors:** Sadat Shafi, Preeti Maurya, Ruby Bansal, Jyoti Sharma, Sai Kumar Mishra, Abul Kalam Najmi, Shailja Singh

**Affiliations:** 1 Department of Pharmacology, School of Pharmaceutical Education and Research, Jamia Hamdard 28848, New Delhi, India; 2 Special Centre for Molecular Medicine, Jawaharlal Nehru University 28754, New Delhi, India; 3 Amity Institute of Virology and Immunology, Amity University 663345, Noida, India; 4 Life Science Research Centre, University of Ostrava 48300 , Ostrava, Czech Republic; The Children's Hospital of Philadelphia, Philadelphia, Pennsylvania, USA

**Keywords:** malaria, drug resistance, *Plasmodium falciparum*, glucose transporter, Ginsenoside Rg1

## Abstract

The emergence of resistance to first-line antimalarial therapies highlights the critical need for next-generation drugs that target distinct molecular pathways and employ novel mechanisms of action. Notably, the intra-erythrocytic parasite development is highly dependent on a sustained glucose supply as their fundamental energy source. Therefore, exploiting a “selective starvation” strategy by targeting the parasite’s reliance on glucose metabolism, particularly through the *Plasmodium falciparum* hexose transporter (*Pf*HT), which is critical for parasite survival, can serve as a promising therapeutic approach to combat multidrug-resistant *Plasmodium* parasites. Through molecular docking and a structure-based drug design approach, we identified a natural compound, Ginsenoside Rg1 (G-Rg1) from the Drug Bank database library, as a potential *Pf*HT inhibitor. The *Pf*HT specificity of G-Rg1 was validated using a yeast complementation model. Subsequently, to investigate the role of *Pf*HT in drug-resistant *Pf* parasites, we investigated the stage-specific expression of *Pf*HT in both artemisinin (ART)-sensitive and resistant *Pf* parasites and reported its elevated expression in resistant parasites, predicting its role in their survival. Notably, *in vitro* growth inhibition studies demonstrated that G-Rg1 effectively suppressed the growth of both ART-sensitive and resistant *Pf* parasites. Additionally, G-Rg1 potentiated the efficacy of dihydroartemisinin in combination and ring survival assays, indicating its potential to circumvent resistance mechanisms. G-Rg1 administration alone and in combination with ART, in *Plasmodium berghei* ANKA-infected mice, reduced parasite multiplication and increased mean survival time. Our findings support G-Rg1 as a promising candidate for drug development against malaria, highlighting the potential of targeting *Pf*HT to combat drug-resistant malaria.

## INTRODUCTION

Despite extensive efforts over several decades to eliminate malaria, it is still a prevalent endemic in the tropical and subtropical subparts of Southeast Asia and sub-Saharan Africa (1). The causative agent responsible for malaria is the *Plasmodium* parasite, which is disseminated through the bite of a female *Anopheles* mosquito, with *Plasmodium falciparum* (*Pf*) listed as the most lethal form (2). Although various classes of drugs, including artemisinin (ART)-based combination therapies (ACTs), have been developed successfully for malaria treatment, nevertheless, the rise of multi-drug-resistant *Plasmodium* strains has created a critical requirement to search and therapeutically investigate new targets to effectively combat this deadly disease (3).

To discover new anti-malarial targets, identifying mechanisms crucial for parasite survival is essential. Notably, glucose represents an important source of energy for *Pf* parasites (4), as they are highly dependent on glycolysis to produce adenosine triphosphate for their energy needs (5, 6). Consequently, *Pf*-infected erythrocytes exhibit approximately a 100-fold increase in glucose acquisition compared to uninfected erythrocytes (7, 8). This metabolic dependence of parasites on glucose highlights metabolic pathways, including the glucose transporter, as an attractive therapeutic target (9). *Pf* hexose transporter (*Pf*HT) serves as the primary glucose transporter in *Pf*, which facilitates the acquisition of this vital nutrient across the plasma membrane of the parasite, ensuring the parasite’s survival (10). Genetic studies have also confirmed *Pf*HT’s essential role in *Plasmodium* species, and it has been independently validated as a promising drug target for malaria treatment (11). Previously, the small-molecule glucose derivative compound 3361 (C3361) has been shown to inhibit *Pf*HT, effectively reducing the growth of intra-erythrocytic *Pf* parasites *in vitro* (12–14). This supports the notion of *Pf*HT as a viable target for anti-malarial drugs. Based on this hypothesis, employing a computational drug discovery approach to screen drug libraries for compounds that bind to *Pf*HT may contribute to the identification of new anti-malarial therapeutics.

In line with this, we screened the Drug Bank database and identified a phytocompound, Ginsenoside Rg1 (G-Rg1), as a high-affinity *Pf*HT binding partner by molecular docking and evaluated its anti-malarial potential in pre-clinical settings.

In order to identify the *Pf*HT specificity of G-Rg1, we used a yeast complementation model system. Like *Plasmodium*, yeast (*Saccharomyces cerevisiae*) requires a constant supply of glucose to grow, and the yeast hexose transporter (*hxt*) is crucial for its glucose uptake. We used a yeast strain (EBY.VW.4000) where all the glucose transporters (*hxt^0^*) have been deleted, limiting its growth on glucose media. Functional complementation of *Pf*HT in *S. cerevisiae hxt* mutant (*hxt*⁰) rescued the growth of yeast significantly but displayed diminished growth in the presence of G-Rg1, which was further correlated with glucose inhibitory potential of G-Rg1 in complemented cells. These results confirmed the *Pf*HT inhibitory effect of G-Rg1. Furthermore, to extend our observations to the role of *Pf*HT in drug-resistant *Pf* parasites, we demonstrated the stage-specific expression of *Pf*HT in ART-sensitive and resistant parasites. Quantitation of mRNA encoding this transporter revealed that its expression is elevated in developmental stages of resistant parasites, thereby indicating it as a potential drug target to combat drug-resistant parasites. Upon experimental validation, G-Rg1 exhibited potent anti-parasitic activity against *Pf* parasites in both *in vitro* assays and *in vivo* mice models. G-Rg1 suppressed the growth of ART-sensitive and resistant parasites within a sub-micromolar concentration, which was confirmed by glucose uptake blocking effect of G-Rg1 in parasites. Moreover, G-Rg1 significantly decreased the ring-stage growth and survival of *Pf* parasites resistant to ART (*Pf*Kelch13^R539T^). Subsequently, G-Rg1 treatment improved the efficacy of ART in inhibiting parasite proliferation, in combination and ring survival assays (RSA), indicating that targeting the parasite’s resistance mechanism is a promising approach to combat the rapid development of drug-resistant parasites. In *in vivo* studies, administration of G-Rg1 alone and/or with ART markedly suppressed the parasite burden and elevated mean survival in infected mice, compared to untreated mice. Overall, our results provide strong evidence for the anti-malarial potential of G-Rg1 and support the therapeutic relevance of targeting *Pf*HT in the development of novel anti-malarial drugs.

## RESULTS

### Ginsenoside Rg1 as a potential binder of *P. falciparum* hexose transporter

**Fig 1:**
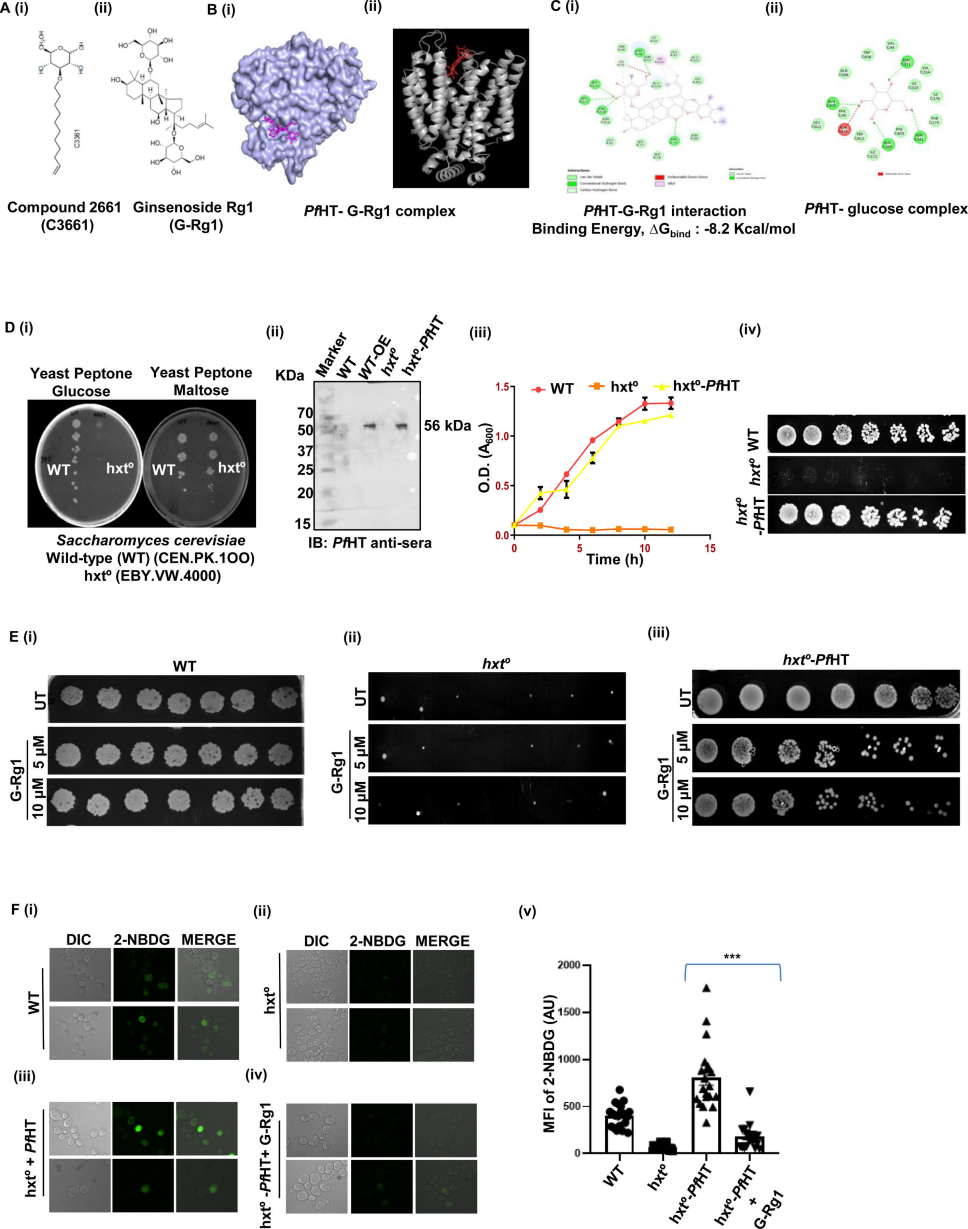
*In silico* interaction study of G-Rg1 with *Pf*HT and effect of G-Rg1 on growth and glucose uptake of *Pf*HT-complemented yeast. (**A**) 2D structure of C3361 (i) and G-Rg1 (ii). (**B**) (i) Possible architecture of the *Pf*HT-G-Rg1 complex. (ii) 3D structure of *Pf*HT1 highlighting the relevant catalytically active site and binding of G-Rg1. (**C**) (i) *In silico* interaction analysis of G-Rg1 with *Pf*HT. G-Rg1 interacts via H-bonds (*n* = 5) with ΔG bind of −8.2 kcal/mol. G-Rg1 primarily engaged with a pocket formed by residues Asn48, Lys51, Thr74, Ser315, Ser317, Asn318, and Glu319. (ii) *In silico* interaction analysis of *Pf*HT in complex with D-glucose indicated that the glucose interacts with residues Ile172, Thr173, Ile176, Gln169, Gln305, Gln306, Ile310, Asn311, and Asn341 and Asn435, which is different from the G-Rg1 binding site. H-bonds. Bond lengths (Å) are highlighted as dashed lines. (**D**) Complementation of *Pf*HT in yeast mutant strain. (i) Culture of WT (CEN.PK2-1C) and *hxt°* (EBY.VW.4000) on yeast peptone dextrose (YPD) and yeast peptone maltose (YPM) plates. (ii) Immunoblot showing the expression of *Pf*HT in WT and mutant yeast strains transformed with *Pf*HT-p416-GPD. Expression was not observed in WT and mutant transformed with only p416GPD. Complementation with *Pf*HT rescues *hxt°* cell growth as depicted using growth curve (iii) and spot assay (iv). Upon complementing *Pf*HT in the *hxt°* strain, restoration of cell growth was observed. (**E**) Effect of G-Rg1 on the growth pattern of *hxt°* strain complemented with *Pf*HT. Spot assay showing growth of yeast cells spotted following 2 days of incubation. Each culture was harvested and adjusted to A_600_ = 0.1 using sterile YPD medium. The particular solution was then serially diluted 10 times, treated with G-Rg1, and each dilution suspension was then spotted on solid YPD medium and incubated for 2 days at 30°C. Interestingly, *Pf*HT-complemented yeast exhibited reduced growth in the presence of G-Rg1 as compared to the control (untreated). However, no effect of G-Rg1 on the growth of WT yeast strain was reported, confirming the *Pf*HT1 specificity of G-Rg1. (**F**) 2-(N-(7-nitrobenz-2-oxa-1,3-diazol-4-yl)amino)-2-deoxyglucose (2-NBDG) uptake assay was used to measure the glucose uptake in yeast cells. *Pf*HT complementation restored the glucose uptake of hxt° cells (i, ii, and iii). G-Rg1 treatment suppressed the 2-NBDG uptake of complemented cells (iv and v) (***, *P* < 0.001).

The molecular docking simulations revealed a distinct binding mode for G-Rg1 within the *Pf*HT binding pocket. Schematic illustration of complex formation between G-Rg1 [2D structure depicted in Fig. 1A (ii)] and the binding pocket of *Pf*HT is shown in Fig. 1B (i) and (ii), respectively. The ligand of interest, G-Rg1, showed a binding energy (∆G_bind_) of −8.2 kcal/mol in its most favorable docked pose. Analysis of the protein-ligand interactions showed that G-Rg1 primarily engages with a pocket formed by residues Asn48, Lys51, Thr74, Ser315, Ser317, Asn318, and Glu319 [Fig. 1C (i)]. Importantly, all of these interactions were characterized by hydrogen bonding, likely contributing to the compound’s strong binding affinity to *Pf*HT.

Interestingly, the binding pocket for G-Rg1 is distinct from the known D-glucose molecular binding pocket of *Pf*HT. The known D-glucose binding site of *Pf*HT consists of interactions with residues Ile172, Thr173, Ile176, Gln169, Gln305, Gln306, Ile310, Asn311, and Asn341 and Asn435 [Fig. 1C (ii)]. This lack of overlap in binding sites suggests that G-Rg1 may not directly compete with glucose for binding to *Pf*HT, but rather may exert its effects through an allosteric mechanism or by inducing conformational modifications in the protein. The strong binding energy of −8.2 kcal/mol for G-Rg1, coupled with its extensive hydrogen bonding network within the binding pocket, provides compelling evidence for its potential as a novel ligand for *Pf*HT.

### *Pf*HT complementation in yeast mutant restores cell growth

For elucidating the functional expression of membrane transporter proteins, including glucose transporters from diverse sources, yeast has proven to be an effective model system. In line with this, Boles and Hollenburg engineered the EBY.VW4000 yeast strain, a hexose transporter-deficient (*hxt^0^*) mutant, in which all hexose transporter-encoding genes, as well as other transporters capable of hexose uptake, have been deleted (15). EBY.VW4000 cannot grow on medium containing just glucose, fructose, or mannose and grows poorly on galactose. For propagation, the *hxt^0^* strain is routinely cultured on maltose, a disaccharide transported via specific maltose symporters (encoded by the *MALx1* loci). Thus, the *hxt^0^* strain provides a perfect platform to clone and characterize hexose transporters from other species, or to replace the function of indigenous transporters. Herein, to validate the functional role of *Pf*HT in *Pf* parasite*s*, we used an orthologous yeast model, *S. cerevisiae* [Fig. 1D (i)]. We effectively complemented the EBY.VW4000 strain with the *Pf*HT. The complementation was confirmed by immunoblotting using *Pf*HT anti-sera [Fig. 1D (ii)]. When the *hxt^0^* yeast strain was complemented with *Pf*HT-p416-GPD, the growth of the complemented yeast re-established in glucose medium as compared to the *hxt^0^* mutant strain, as shown by the growth curve and spot assays [Fig. 1D (iii) and (iv)]. CEN.PK2-1C was used as a wild-type (WT) control. The *Pf*HT-p416 GPD complemented strain was used to identify the *Pf*HT inhibitory potential of G-Rg1.

### Ginsenoside Rg1 suppressed growth of complemented yeast by inhibiting *Pf*HT

Having validated the role of *Pf*HT complementation in rescuing the growth of mutant yeast cells in the presence of glucose, the next objective was to determine whether G-Rg1 could specifically target *Pf*HT in the yeast complementation model. To address this, CEN.PK2-1C (WT), *hxt^0^*, and *Pf*HT complemented yeast strain (*hxt^0^-Pf*HT-p416) was cultured in the presence and absence of G-Rg1 for 12 h to assess growth patterns. Notably, spot assay analysis revealed that *hxt^0^-Pf*HT-p416 complemented yeast mutants exhibited reduced growth upon G-Rg1 treatment (Fig. 1E). This effect is likely attributable to the drug-targeting activity of G-Rg1 against *Pf*HT.

### Ginsenoside Rg1 reduced the glucose uptake of *Pf*HT1 complemented yeast cells

Glucose uptake in yeast cells and the modulatory impact of G-Rg1 on *Pf*HT complemented cells was quantitatively assessed using the fluorescent glucose probe, 2-(N-(7-nitrobenz-2-oxa-1,3-diazol-4-yl)amino)-2-deoxyglucose (2-NBDG). Complementation of *Pf*HT in *hxt^0^* yeast restored the glucose uptake capacity, as evidenced by increased 2-NBDG fluorescence intensity [Fig. 1F (i), (ii) and (iii)]. However, exposure of *Pf*HT-complemented cells to G-Rg1 led to a marked decrease in 2-NBDG uptake [Fig. 1F (iv)], indicating a significant inhibitory effect on glucose transport activity [Fig. 1F (v)].

### Expression of *Pf*HT in intra-erythrocytic drug-sensitive and resistant parasites

**Fig 2:**
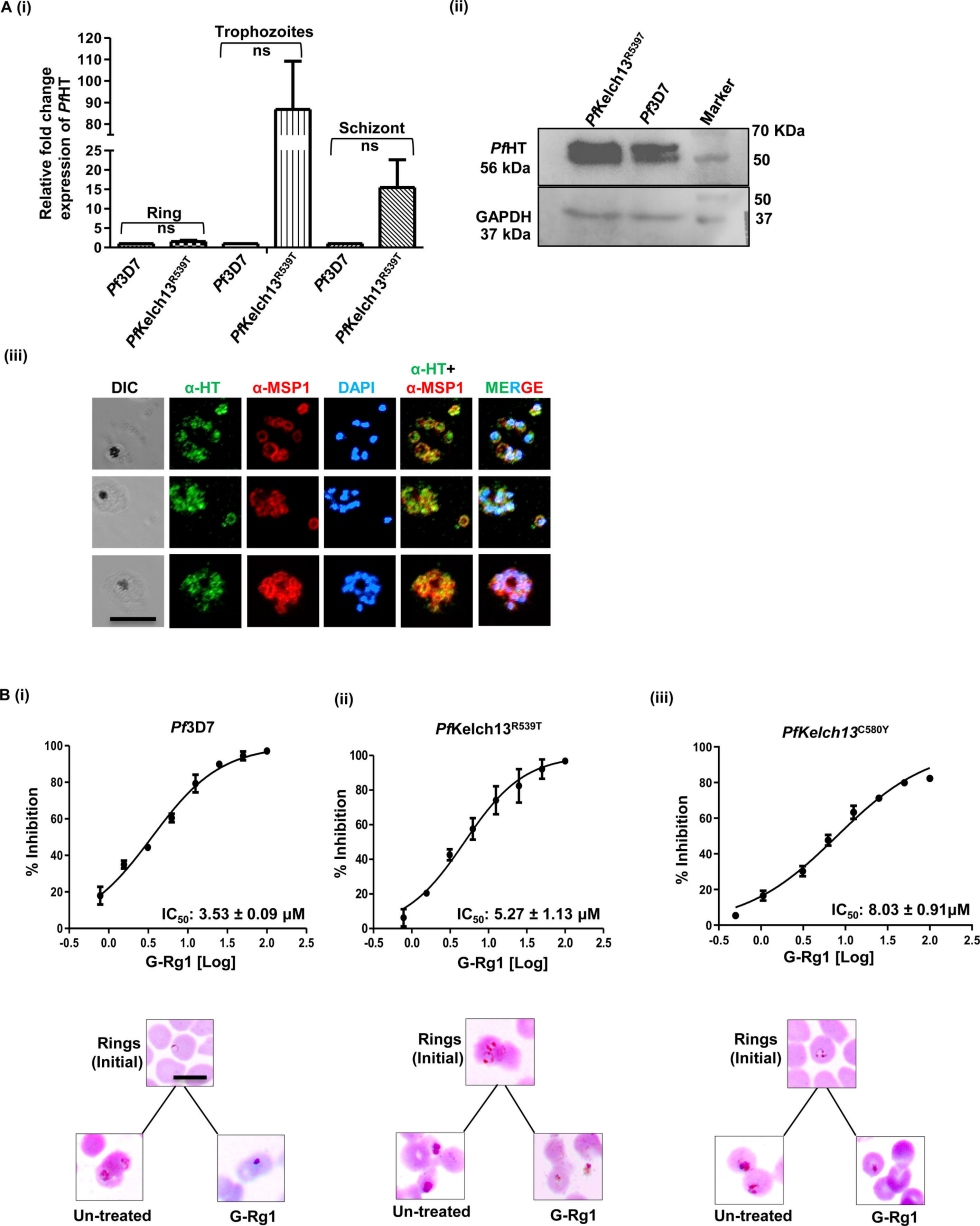
Expression and localization of *Pf*HT and effect of G-Rg1 on growth and progression of ART-sensitive and resistant strains of *P. falciparum* parasites*.* (**A**) (i) Quantitative RT-PCR analysis of *Pf*HT in cDNA derived from ring, trophozoite, and schizont stages of *Pf*3D7 and *Pf*Kelch13^R539T^. 18S rRNA transcripts were taken as a loading control. (ii) Immunoblot using *Pf*HT antisera GAPDH antisera showing *Pf*HT1 (~56 kDa) and GAPDH in mixed parasite lysates of *Pf*3D7 and *Pf*Kelch13^R539T^. (iii) Localization of *Pf*HT within mature schizont: green represents *Pf*HT, red represents Merozoite Surface Protein-1 (MSP-1). DIC represents differential interference contrast. Parasite nuclei were stained with DAPI. Immuno-fluorescence assay results depict that *Pf*HT is localized to the plasma membrane of the parasite. (**B**) Graphs representing concentration-dependent growth inhibitory effect of G-Rg1 on ART-sensitive *Pf*3D7 (i) and resistant *Pf*Kelch13^R539T^ (ii) and *Pf*Kelch13^C580Y^ (iii) strains, as evaluated by scoring Giemsa-stained parasites. The corresponding Giemsa-stained images of parasites pre- and post-66 h treatment at corresponding IC_50_ ± SEM values of G-Rg1 showed the formation of pyknotic bodies (scale bar = 2 µm). The result depicts the mean of three independent experiments.

The expression of *Pf*HT was demonstrated both at the RNA and protein levels. Stage-specific transcript levels of *Pf*HT in *Pf*3D7 and *Pf*Kelch13^R539T^ were detected by qPCR utilizing cDNA obtained from tightly synchronized cultures of rings, trophozoites, and schizont-stage infected erythrocytes of both the strains. *Pf*HT encoding transcripts were amplified using specific primers which revealed higher expression of *Pf*HT in the drug-resistant *Pf* parasites, specifically at mature (trophozoite and schizont) stages, compared to sensitive counterparts, predicting a role of *Pf*HT in the survival of resistant parasites [Fig. 2A (i)]. 18S served as a housekeeping control to ensure equal loading of RNA from each developmental stage for RT-qPCR analysis.

Immunoblot analysis was employed to evaluate the expression of native *Pf*HT in parasite lysates from the *Pf*3D7 and *Pf*Kelch13^R539T^ strains by using mice raised anti-*Pf*HT antibodies (Miscellaneous, S1). Lysates were produced from a mixed-stage population of parasites, and distinct bands corresponding to *Pf*HT were found at the predicted molecular weights in both strains. A prominent band of about 56 kDa was seen, which corresponds to the anticipated molecular weight of the full-length *Pf*HT protein in *Pf* [Fig. 2A (ii)]. GAPDH served as a loading control.

### *Pf*HT localizes to the *Plasmodium* surface and colocalizes with MSP-1

To determine the cellular localization of *Pf*HT within the *Pf* parasite, fixed-late stage parasite smears were immunostained with *Pf*HT antisera along with Merozoite Surface Protein-1 (MSP-1) antisera, a well-established marker of the merozoite surface. *Pf*HT was observed to be localized on the merozoite surface in punctated schizonts. Co-localization with MSP-1 (GPI-anchored membrane protein) indicates that *Pf*HT is localized at the parasite plasma membrane [Fig. 2A (iii)].

### Ginsenoside Rg1 suppressed the growth of intra-erythrocytic *P. falciparum* parasites

The *in vitro* treatment of ART-sensitive (*Pf*3D7) and resistant (*Pf*Kelch13^R539T^, *Pf*Kelch13^C580Y^) parasites with G-Rg1 demonstrated growth suppression with IC_50_ ± SEM values of 3.53 ± 0.09, 5.27 ± 1.13, and 8.03 ± 0.91 µM, respectively [Fig. 2B (i), (ii) and (iii)]. The IC50 values of resistant stages were significantly higher than the sensitive parasites, indicating that reduced drug sensitivity is associated with the resistant phenotype. This finding is consistent with an increased functional role of *Pf*HT in resistant parasites, which may contribute to the reduced inhibitory effect of G-Rg1. Furthermore, analysis of parasite development at 66 h post-treatment revealed that G-Rg1 impaired maturation into trophozoites in both drug-sensitive and resistant strains, instead inducing the formation of pyknotic bodies.

### Ginsenoside Rg1 impeded the growth of *P. falciparum* parasites maximally at the mature stages

**Fig 3:**
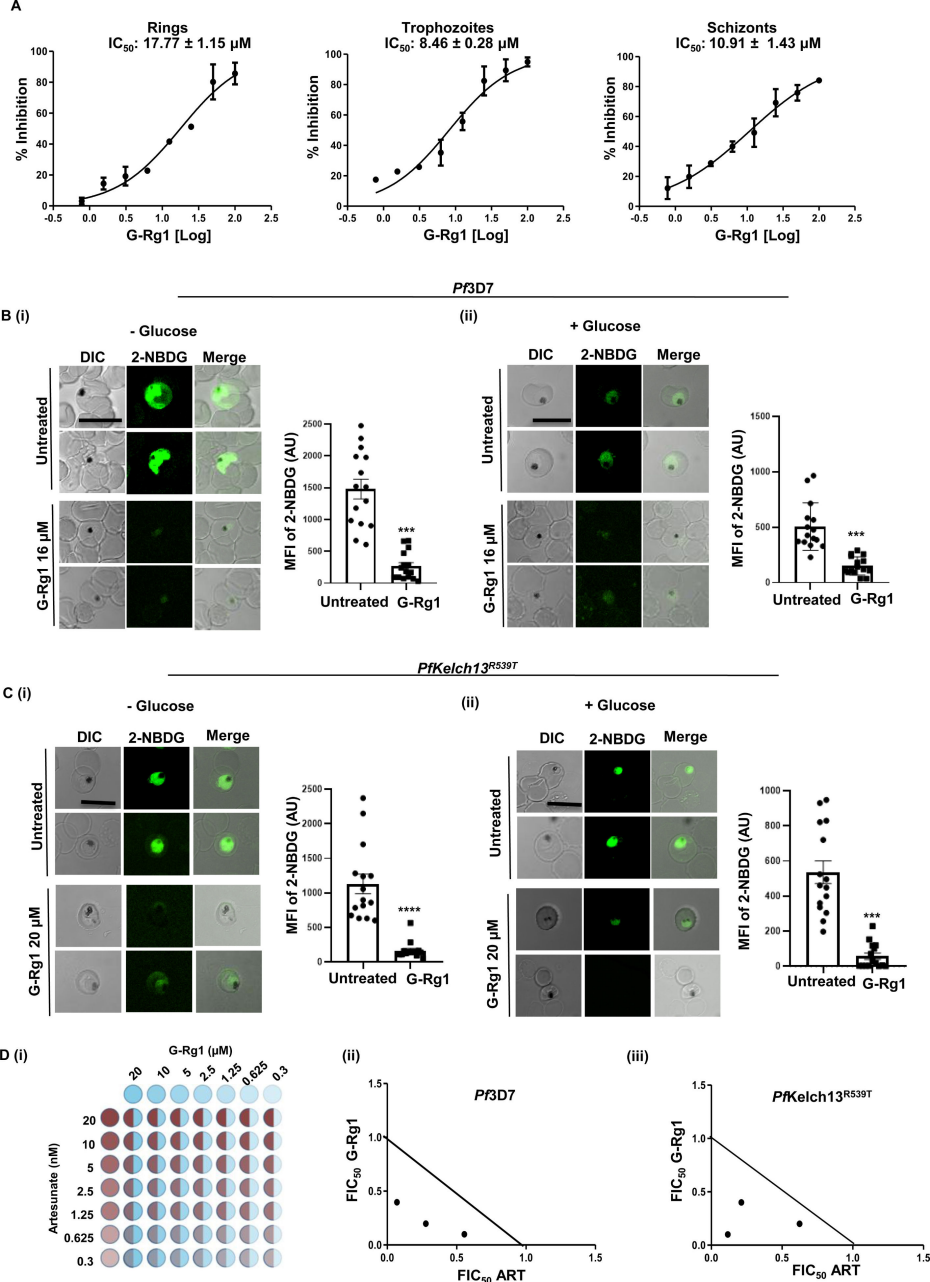
Stage-specific growth inhibitory and glucose uptake blocking potential of G-Rg1 and its combinatorial effect with artesunate. (**A**) The graphs depicting the percent reduction in parasitemia by G-Rg1 at each parasitic development stage, along with the respective stage-specific IC_50_ ± SEM values. The result depicts the mean of three independent experiments. (**B**) G-Rg1 significantly inhibited the glucose uptake, both in the presence (i) and absence (ii) of glucose in *Pf*3D7 parasites**.** (**C**) A similar glucose inhibitory effect of G-Rg1 was also reported in *Pf*Kelch13^R539T^ (i and ii) parasites as assessed by 2-NBDG fluorescence measurement using confocal microscopy. (**D**) Combinatorial growth inhibitory effect of G-Rg1 and artesunate on *Pf*. Schematic representation of drug treatment using checkerboard assay (i). Iso-bolograms depicting interaction between G-Rg1 and ART against (ii) ART-sensitive (*Pf*3D7) and (iii) resistant (*Pf*Kelch13^R539T^) strains of *Pf*. Mean FIC_50_ of ART and G-Rg1 were taken on the x- and y-axis, respectively. Results are representative of three independent experiments (***, *P* < 0.001, ****, *P* < 0.0001).

During intra-erythrocytic development of *Pf*, the parasite has to undergo a progression through several stages, i.e., rings, trophozoite, and schizonts. The growth-inhibitory capacity of G-Rg1 on various developmental stages was assessed using an *in vitro* stage-specific inhibition assessment. Parasites (tightly synchronized at every stage) were treated with G-Rg1 for 6 h, subsequently washed with iRPMI after every treatment period, resulting in parasites progressing in the absence of the drug for 66 h. A brief exposure (6 h) of parasites to G-Rg1 at different developmental stages demonstrated a stage-dependent chemo-suppressive effect (Fig. 3A). The IC₅₀ ± SEM value at the ring stage was significantly higher (17.77 ± 1.15 µM) compared with those observed at the trophozoite (8.46 ± 0.28 µM) and schizont (10.91 ± 1.43 µM) stages, with statistical analysis confirming significance (*P < 0.05). These findings indicate that later intra-erythrocytic stages are more susceptible to G-Rg1 than ring-stage parasites. The increased efficacy of G-Rg1 at later stages can be linked with its highly active metabolic activity with increased glucose acquisition.

### Ginsenoside Rg1 reduced the glucose uptake of parasites

The effect of G-Rg1 on glucose acquisition of parasites was evaluated using 2-NBDG. Treatment with G-Rg1, under both glucose supplemented and glucose-deprived conditions, significantly suppressed 2-NBDG uptake in drug-sensitive [Fig. 3B (i) and (ii)] and resistant parasites [Fig. 3C (i) and (ii)], thereby confirming its anti-malarial effect via inhibiting glucose transport. Depriving the parasites of their prime energy source can disrupt their metabolic signaling, resulting in cell death. These findings strongly suggest that G-Rg1 selectively inhibits *Pf*HT, leading to impaired growth, potentially due to restricted glucose uptake, thereby disrupting metabolic homeostasis.

### Ginsenoside Rg1 and artesunate synergistically inhibited the growth of *Pf* parasites

Combination therapy is an effective strategy currently employed to tackle resistant malaria, involving the use of two or more therapeutic agents in conjunction. A checkerboard combination assay was used to determine the nature of *in vitro* interaction of G-Rg1 and ART on parasite growth. A synergistic effect was observed in the *Pf*3D7 strain at some concentrations, with an FIC₅₀ index of <0.5 [Fig. 3D (ii)]. Similarly, in ART-resistant parasites, G-Rg1 significantly enhanced ART efficacy, demonstrating a comparable synergistic interaction [Fig. 3D (iii)]. Notably, G-Rg1 potentiated ART activity in both ART-sensitive and resistant parasites, highlighting its potential as a combination partner for ART.

### Ginsenoside Rg1 enhanced antimalarial potency of dihydroartemisinin against resistant parasites

**Fig 4:**
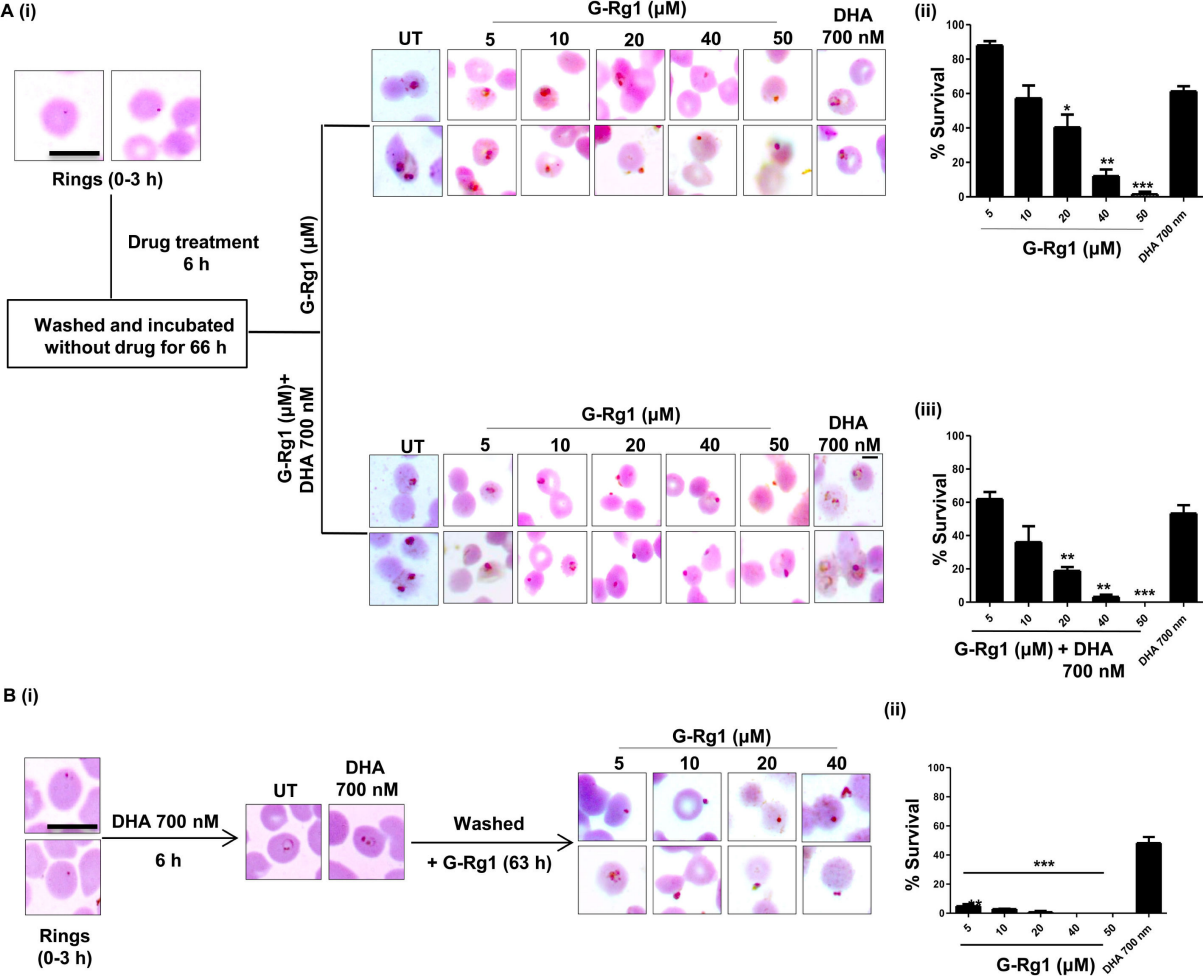
G-Rg1 reduced the survival of DHA-resistant ring stage resistant parasites alone and in combination with DHA. (**A**) (i) Schematic representation showing light micrographs of Giemsa-stained infected RBCs incubated with DHA and G-Rg1 alone, as well as in combination with DHA. Bar graphs representing percentage survival following treatment with DHA and G-Rg1 alone (ii) and in combination (iii). The combination of G-Rg1 with DHA alleviates the antiparasitic activity of DHA against ring stages of ART-resistant parasites. (**B**) (i) Schematic diagram showing the modified form of RSA, wherein ART-resistant ring-staged parasites were treated with DHA, followed by treatment with various concentrations of G-Rg1. The surviving parasites were scored, and the percentage survival was calculated for each condition. (ii) G-Rg1 potently decreases the survival of DHA-pretreated resistant rings when compared to DHA alone. Results represent the mean of three independent experiments (*, *P* < 0.05, **, *P* < 0.01, ***, *P* < 0.001).

RSA is a reference assay to measure ART resistance against *Pf in vitro* setting. ART resistance is correlated with the reduced susceptibility of ring-stage parasites to DHA leading to delayed parasite clearance with ART monotherapy or 3-day ACT course in clinic. We evaluated the efficacy of G-Rg1 alone and in combination with DHA on ART-resistant (*Pf*Kelch13^R539T^) parasites. Early ring-stage parasites, treated with DHA 700 nM, various concentrations of G-Rg1 alone, as well as in combination with DHA 700 nM, exhibited normal progression of untreated parasites to mature trophozoites, whereas G-Rg1 and DHA-treated cultures displayed unhealthy and dead parasites [Fig. 4A (i)]. Following incubation of 66 h, a significant reduction in parasite survival was observed in G-Rg1 treated parasites as compared to DHA-treated ones. Notably, G-Rg1 treatment at 40 μM (8×IC₅₀) and 50 μM (10×IC₅₀) led to over 85% reduction in parasite survival, compared to a 48% reduction with DHA (700 nM) [Fig. 4A (ii)]. Notably, G-Rg1 and DHA in combination increased the potency of DHA by lowering parasitemia by 70%–95% at different G-Rg1 concentrations [Fig. 4A (iii)]. We further evaluated the inhibitory efficacy of G-Rg1 against resistant parasites that survived DHA treatment [Fig. 4B (i) and (ii)]. The percentage of parasite survival (DHA-pretreated rings) reduced in the presence of G-Rg1, indicating that G-Rg1 is powerful in clearing resistant parasites that survive post-DHA treatment, thus predicting it as a potential partner candidate for combination therapy.

### Ginsenoside Rg1 impeded parasite growth *in vivo*

**Fig 5:**
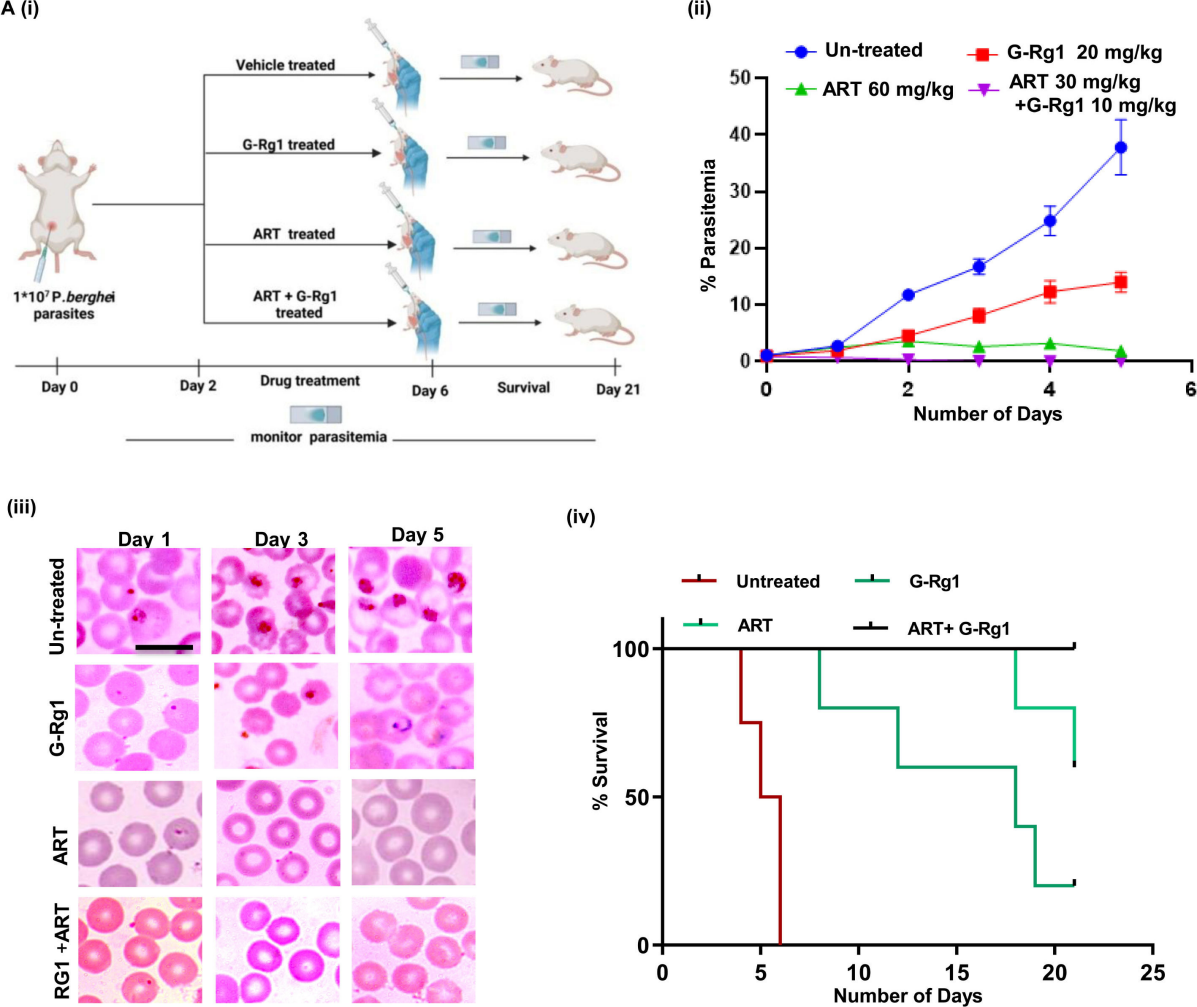
Effect of G-Rg1 on parasite growth in the *in vivo* malaria model. (**A**) (i) Schematic representation of malaria induction and *in vivo* drug treatment regimen, vehicle-treated mice (*n* = 5) served as a control. (ii) Graph showing routine percent parasitemia of untreated, G-Rg1-treated, ART-treated, and combination-treated infected mice. (iii) Micrographs of Giemsa-stained blood smears of control and treated mice, indicating parasite load in mice from each group**.** (iv) Plot showing the survival of untreated and drug-treated mice as observed for up to 21 days post-infection. G-Rg1, alone as well as in combination, significantly reduced the parasite burden and increased the survival of infected mice.

*In vivo* antiplasmodial activity of G-Rg1 alone, as well as in combination with ART, was demonstrated using *Plasmodium berghei* ANKA-infected mice model (*n* = 5 mice/group) [Fig. 5A (i)]. Infected mice were orally administered G-Rg1 (20 mg/kg), ART (60 mg/kg), G-Rg1 (10 mg/kg) + ART (30 mg/kg), and phosphate-buffered saline (PBS) as a vehicle control for five consecutive days. The blood smears from the tail vein were made regularly to estimate the parasitemia. G-Rg1-treated mice exhibited a 60% decrease in parasite burden compared to the vehicle control [Fig. 5A (ii) and (iii)]. Additionally, mice treated with G-Rg1 exhibited a survival duration exceeding 15 days, in contrast to vehicle-treated controls, which succumbed within 10 days following infection [Fig. 5A (iv)]. Notably, the combination of G-Rg1 and ART prominently suppressed parasite burden and extended the mean survival rate of infected mice. Mice receiving the combination therapy (at half doses of each drug) were alive even after 21 days. These preclinical findings highlight G-Rg1 as a potential partner drug for ART.

## DISCUSSION

The increasing prevalence of drug-resistant *Plasmodium* strains poses a significant challenge in malaria treatment (16), underscoring the need for investigation of new drug targets, as well as innovative therapeutic strategies (17, 18). *Pf*HT has emerged as a promising and highly selective therapeutic target to combat *Pf* infection (19, 20). As an essential membrane protein responsible for glucose uptake, *Pf*HT is the parasite’s sole facilitative glucose transporter throughout its intra-erythrocytic developmental stages (7). In contrast to the multiple redundant glucose transporters in human cells, *Pf* relies exclusively on *Pf*HT, making it uniquely vulnerable to targeted inhibition (7, 9). Notably, phytochemicals represent a promising therapeutic lead for new antimalarial development. They have long served as a valuable source of bioactive compounds, as most of the anti-malarials currently in use or historically significant trace their origins to natural products derived from plants (21). In line with this, our study identified a natural compound, G-Rg1, as a potential inhibitor of *Pf*HT, through an *in silico* SBDD approach, and validated its *Pf*HT inhibitory potential using a yeast complementation model. These complementation models offer a unique platform for functionally dissecting *Pf* membrane transporters and screening for selective inhibitors (22, 23). Their simplicity, scalability, and specificity make them indispensable for preclinical antimalarial drug discovery pipelines, particularly when targeting essential nutrient or drug transporters like *Pf*HT (24, 25). Expression of *Pf*HT (codon-optimized) in *S. cerevisiae* mutant (EBY4000) yeast cells, that are null mutants for their endogenous hexose transporters (*hxt*) (15), restored their growth in glucose media, with respect to mutant (*∆hxt*) cells. Study results (growth curve, spot assay, and 2-NBDG uptake) revealed that the growth of the complemented line was completely dependent upon uptake of glucose through *Pf*HT, confirming substrate specificity of complemented cells. *Pf*HT selectivity of G-Rg1 was also determined using the same complemented system. Interestingly, we observed that G-Rg1 decreased the growth and glucose uptake of *Pf*HT-complemented yeast cells, providing evidence for its specificity to *Pf*HT.

Furthermore, the expression of *Pf*HT was validated at both the transcript and protein levels during the intra-erythrocytic stages of the *Pf* parasite, using quantitative RT-PCR and immunoblotting. Interestingly, stage-specific mRNA expression results revealed an increased expression of *Pf*HT1 in resistant *Pf* parasites (*Pf*Kelch13^R539T^) compared to drug-sensitive (*Pf*3D7) ones. This observation highlights a potentially underappreciated facet of resistance biology, namely, the contribution of metabolic adaptations to drug tolerance and survival (26). The elevated expression of *Pf*HT observed in resistant parasites suggests a compensatory mechanism wherein parasites increase glucose transporter expression to sustain energy production under drug-induced stress. From a therapeutic perspective, these findings reinforce the rationale for targeting parasite metabolism in resistant *Plasmodium* parasites.

In line with this, our study results reported that G-Rg1 exhibited significant *in vitro* growth inhibitory effects on drug-sensitive (*Pf*3D7) and resistant (*Pf*Kelch13^R539T^, *Pf*Kelch13^C580Y^) parasites, with IC_50_ values of 3.53 ± 0.09, 5.27 ± 1.13, and 8.03 ± 0.91 µM, respectively. These results are in line with an increased functional role of *Pf*HT in resistant parasites, which may contribute to the reduced inhibitory effect of G-Rg1. Notably, G-Rg1 had the capacity to decrease glucose absorption by parasites, both in the presence and absence of glucose, as determined by 2-NBDG uptake assay. Our findings align with the previous studies that emphasized that targeting glucose transport in parasites can starve them, leading to reduced growth and proliferation (12, 19).

The combination of *Pf*HT inhibitors with ART represents a rational and promising strategy to counteract ART resistance by exploiting the parasite’s metabolic vulnerabilities. As drug resistance continues to rise, such metabolism-targeted combinations may provide a vital tool for sustaining the efficacy of antimalarial therapies. Interestingly, we observed a synergistic anti-malarial association between G-Rg1 and artesunate in both strains, as determined by the checkerboard combination assay.

Clinically, ART partial resistance is characterized by a half-life of ≥5 h in peripheral blood following treatment. RSA is regarded as the gold standard assay for assessing ART resistance in *in vitro* settings (27). This assay requires tight synchronization of parasites to the 0–3 h ring stage, a critical window that enables discrimination between ART-sensitive and ART-resistant strains (28). We employed RSA to evaluate the effect of G-Rg1 on the ART-resistant *Pf* parasites (*Pf*Kelch13^R539T^). Our results demonstrated that G-Rg1, both as a monotherapy and in combination therapy with DHA, potently suppressed the growth of ART-resistant parasites. Furthermore, G-Rg1 significantly eliminated parasites that had survived prior DHA exposure. These findings further highlight the role of *Pf*HT in the survival of ART-resistant parasites.

Evaluating the *in vivo* efficacy of a compound is vital for the identification of promising lead candidates during the preclinical stage of drug development. We evaluated the *in vivo* anti-malarial activity of G-Rg1 in *P. berghei*-infected mice. G-Rg1 treatment alone and in combination with ART reduced the parasitic load, as well as enhanced the survival of the mice. Altogether, our study not only advanced our understanding of *Pf* metabolism but also opened new avenues for the development of antimalarial therapies. Additionally, exploring the drug’s effects on other stages of the *Pf* lifecycle may reveal broader applications in malaria treatment. This study supports ongoing efforts to manage drug resistance and mitigate the global burden of this life-threatening disease.

## MATERIALS AND METHODS

### Screening of drug library for a potential inhibitor of *Pf*HT

By utilizing an *in silico* therapeutic repositioning method (29), we screened the Drug Bank database (https://go.drugbank.com) of pharmacologically active compounds for similarities with the specific *Pf*HT inhibitor, C3361. Notably, C3361, a glucose analog [Fig. 1A (i)], has been shown to moderately inhibit *Pf*HT and impede the survival and proliferation of intra-erythrocytic parasites *in vitro* (12, 14). We retrieved Structural Data Format (SDF) files for C3361 and the Drug Bank compounds from PubChem, which provides open access to chemical information and biological activity data (30). We performed structural superimposition of the drug bank library compounds with C3361 using Discovery Studio Visualizer v20.1.0.19295, generated by Dassault Systèms Biovia Corp., to evaluate structural similarity for each of the compounds with C3361 as a reference.

### *In silico* interaction analysis of Ginsenoside-Rg1 with *Pf*HT

*In silico* molecular docking analysis was performed to investigate the binding interactions between the *Pf*HT and G-Rg1. SDF file of G-Rg1 was downloaded from PubChem (31) and SDF format was changed to standard PDB format, subsequently followed by production of its energy-minimized 3D structure using Chem3D Pro 12.0, as described earlier. The crystal structure of *Pf*HT (PDB ID: 6M20) (32) was retrieved from the Protein Data Bank and prepared for docking using AutoDock Tools. AutoDock Vina was employed for the docking simulations due to its efficient search algorithm and accurate scoring function (33). A blind-docking approach was chosen to determine similarity in the docking sites of G-Rg1 and glucose. A grid box encompassing the entire *Pf*HT protein was defined. The dimensions and center of the grid box were adjusted to include all potentially interacting residues. Docking parameters were optimized to balance computational efficiency and thoroughness, with the exhaustiveness set to 8 and the number of output poses set to 9. Post-docking analysis was performed using Discovery Studio Visualizer. The best-scoring poses for each ligand were selected based on binding energy and visual inspection of the protein-ligand interactions. Interacting residues were identified through the molecular interactions in the binding pocket. Two-dimensional interaction diagrams were generated for G-Rg1 and D-glucose to visualize key protein-ligand contacts, including hydrogen bonds, hydrophobic interactions, and π-stacking.

### Generation of *Pf*HT-complemented yeast strain

The complementary determining sequence that encodes *Pf*HT was codon-optimized for expression in yeast (GenScript Biotech, United States). *Pf*HT complementation in hexose transporter mutant *S. cerevisiae* strain: EBY.VW.4000 (*hxt°*) (34) was performed by transforming codon-optimized *Pf*HT-p416-GPD construct and p416-GDP (vector only) in this strain using Frozen-EZ Yeast Transformation IITM kit (Zymo Research) in accordance with manufacturer’s protocol, and plated on yeast nitrogen base agar plate (supplemented with 2% glucose and/or maltose and 1× amino acid mix without uracil) as selective media, and cells were cultivated at 30°C. *S. cerevisiae* yeast strain CEN.PK2-1C served as the WT control. The complementation of *Pf*HT-p416-GPD was further confirmed by immunoblotting using in-house generated *Pf*HT specific primary antibody.

### Growth analysis in *Pf*HT complemented yeast

To evaluate the growth dynamics of *Pf*HT complemented yeast mutant strain, we pre-cultured WT, *hxt°,* and *hxt°-Pf*HT1-p416-GPD strains in yeast peptone dextrose (YPD) media (1% yeast extract, 2% glucose, and 2% peptone) at 30**°**C. Subsequently, the pre-culture was diluted, using YPD media, to an optimum optical density (A_600_) of 0.1 before being employed as the primary culture. Every 2 h, the A_600_ was quantified spectrophotometrically to determine the growth of WT and complemented yeast cells (35). Furthermore, after 24 h of incubation period, each culture was collected and corrected to A_600_ = 0.1 using YPD media. These solutions were then serially diluted ten times, and each diluted mixture was placed on YPD solid plates and cultured for 2 days at 30°C.

### Assessment of Ginsenoside-Rg1 selectivity for *Pf*HT

To evaluate the specificity of G-Rg1 (Sigma Aldrich, USA) toward *Pf*HT, WT, *hxt°,* and *hxt°-Pf*HT1-p416 strains were grown overnight in YPD media until adequate growth was accomplished. Consequently, yeast cultures were diluted with sterile YPD medium to an optical density (A_600_) of 0.1 and maintained in the presence or absence of 5 and 10 μM G-Rg1 for 12 h at 30°C. After incubation, the cultivated cell suspension was serially diluted 10-fold and spotted onto YPD agar plates and cultured at 30°C for 2 days (36).

### Parasite culture

*Pf* laboratory-adapted strains *Pf*3D7, *Pf*Kelch13^R539T^, and *Pf*Kelch^C580Y^ were cultured *in vitro* in accordance with the standard protocols of Trager and Jansen (37). Briefly, parasites were cultured in O+ erythrocytes (Rotary Blood Bank, New Delhi) at 2% hematocrit in RPMI 1640 medium (Gibco, USA), supplemented with Sodium bicarbonate (2 g/L, Sigma Aldrich, USA), Hypoxanthine (50 mg L^−1^, Sigma Aldrich, USA), Albumax I (0.5%, Invitrogen, USA), and Gentamicin (10 mg/L, Sigma Aldrich, USA), at 37°C in a mix gas setting (90% N_2,_ 5% CO_2_, and 5% O_2_). Parasite culture was regularly monitored by preparing thin smears, fixing them with methanol (Merck), and staining with Giemsa solution (5%, Sigma Aldrich, USA). Giemsa-stained parasites were visualized using a compound microscope (Olympus, Tokyo) with oil immersion at 100× magnification. Synchronization of parasites at the ring stage was obtained by treating them with sorbitol (5%, Sigma Aldrich) in two successive cycles, following established protocol (38).

### Stage-specific analysis of *Pf*HT using RT-PCR

Stage-specific expression of *Pf*HT was confirmed through reverse transcription qPCR (39). The cDNA from synchronous cultures of *Pf*3D7 and *Pf*Kelch13^R539T^ strains at the ring, trophozoite, and schizont stages was used to amplify transcripts corresponding to *PfHT* and *18S* rRNA genes using the following set of primers, *Pf*HT: forward 5′-GGTGCTGTGTTAGGATGTGGT-3′; and, reverse 5′-ACACCATACGCACCCTTCTT-3′, and *Pf*18S: forward 5′-CCGCCCGTCGCTCCTACCG-3′; and, reverse 5′-CCTTGTTACGACTTCTCCTTCC-3′. The resulting data were analyzed using Step-One software (Applied Biosystems) by calculating the comparative Ct values for the reactions.

### Detection of *in vivo* expression of *Pf*HT in parasites

To evaluate *Pf*HT expression in *Pf* parasites, late-stage *Pf*3D7 and *Pf*Kelch13^R539T^ parasites were extracted using saponin (5%, Sigma Aldrich, USA) (40). The resulting parasite pellet was lysed with RIPA buffer (Thermo Scientific, USA) supplemented with protease inhibitor. The lysate was then resolved using SDS-PAGE (12%), followed by transfer onto a nitrocellulose membrane. The blot was probed with *Pf*HT anti-sera diluted to 1:500 for 1 h at room temperature (RT), followed by probing with an HRP-conjugated anti-mice secondary antibody (1:5,000; Sigma-Aldrich) at RT for 1 h.

Immuno-fluorescence assay was conducted on *Pf*3D7 parasites to detect the localization of *Pf*HT in *Pf* parasite, as described earlier (39). The mature-stage parasite smears were probed with anti-*Pf*HT (1:200) and anti-MSP-1 antibody (1:300), followed by incubation with secondary antibodies (anti-mice Alexa Fluor 488 and anti-rabbit AF 546). The slides were treated with DAPI antifade (Invitrogen) and pictographed using a confocal microscope (NIKON Corporation).

### Growth inhibition assay

To determine the anti-malarial potential of G-Rg1 and to assess its half-maximal drug inhibitory concentration values (IC_50_) against *Pf*3D7, *Pf*Kelch13^R539T^, *Pf*Kelch13^C580Y^ asexual blood stage parasites, synchronized *Pf*-infected ring stage erythrocyte cultures at a parasitemia of 0.8% and a hematocrit of 2% were incubated with different concentrations (50–0.58 μM) of G-Rg1 for 66 h (41). Untreated and positive (artesunate, Sigma Aldrich, USA) controls were cultivated and processed under identical conditions. To assess the inhibitory effect of G-Rg1 on parasite growth, parasitemia was estimated by manually scoring the number of infected cells in an approximated 2,000 erythrocytes, and % inhibition was computed relative to the untreated control employing this formula. Experiments were performed as three biological replicates, each *N* = 3.


$$\%\;\text{Inhibition}=100\times \frac{(\%\;Parasitemia\;of\;Control-\%\;\text{Parasitemia}\;of\;Treated)}{\%\;\text{Parasitemia}\;of\;Control}$$

The IC_50_ values of G-Rg1 in *Pf*3D7, *Pf*Kelch13^R539T^, and *Pf*Kelch13^C580Y^ were estimated by plotting percent inhibition values versus log concentration of compound using GraphPad PRISM software.

### Stage-specific growth inhibition assay

The stage-specific growth inhibitory effect of G-Rg1 on *Pf* parasites was evaluated following a standardized protocol (42). Briefly, synchronized parasites (*Pf*3D7) at 0.8% parasitemia were subjected to different concentrations of G-Rg1 (for 6 h) at ring stage (6 to 12 h), trophozoite stage (28 to 34 h), and schizont stage (42 to 48 h). After the 6-h incubation, G-Rg1 was eliminated by washing the culture with complete medium, and the parasites were cultured further till the trophozoite stage of the next cycle. Untreated parasites were treated as a control. The percentage of chemo-suppression was determined by comparing the parasitemia levels of drug-treated parasites with those of the untreated control. Experiments were performed as three biological replicates, each *N* = 3.

### 2-NBDG uptake assay

Glucose uptake by yeast cells and *Pf* parasites was quantified using 2-NBDG, a fluorescent D-glucose analog (Sigma-Aldrich, USA), using confocal microscopy. For yeast, cells cultured in glucose-free medium to mid-log phase (A_600_ of 1) were harvested and re-cultured in the above-mentioned media and incubated with 2-NBDG (60 μM) for 30 min at 30°C. Subsequently, *Pf*HT complemented cells were treated with G-Rg1 (10 μM) for 6 h and incubated with 2-NBDG for 30 min. To end the 2-NBDG absorption process, the incubation mixture was removed, and the cells were washed thrice with 1× phosphate-buffered saline. Live cells were visualized and imaged with a NIKON confocal microscope and 2-NBDG fluorescence was quantified using NIS elements software. Mean fluorescence intensity was determined for individual cells within the imaging field, and values were normalized by subtracting the background signal measured from a cell-free region to account for non-specific fluorescence (43). At least 15–20 cells were analyzed from three independent experiments, and data were plotted as means ± SEM.

Similarly, synchronized early trophozoite stage parasites were treated with G-Rg1 (4× IC_50_), in the presence and absence of glucose, and kept for 6 h at 37°C. The culture media were exchanged with RPMI (without glucose) supplemented with 2‐NBDG (100 µM), accompanied by incubation at 37°C for 30 min to facilitate the absorption of the glucose analog (44). To end the 2-NBDG absorption process, the incubation mixture was removed, and the cells were washed with RPMI media twice, followed by washing with PBS and processed in a similar manner as mentioned above.

### Drug combination assay

The combinatorial effect of G-Rg1 and ART on *Pf* parasites was demonstrated in accordance with the checkerboard assay (45). In this experiment, two drugs are evaluated in double serial dilutions, and the effect of each drug is determined both as monotherapy and in combination. The interaction between the two drugs is then demonstrated either geometrically or algebraically. In brief, ring-staged *Pf* parasites (*Pf*3D7 and *Pf*Kelch13^R539T^) at 0.8%–1% parasitemia were exposed to varying concentrations of G-Rg1 (0.3–20 µM) and ART (0.3–20 nM), both individually and in combination, for 66 h following the checkerboard assay protocol. Untreated parasites were marked as controls. Fractional inhibition concentrations (FICs) were calculated as reported earlier, with FIC values <0.5 indicating a synergistic interaction between the two compounds. Experiments were performed in a replicate of three, each *N* = 3.

### Ring survival assay

To determine the efficacy of G-Rg1 on *Pf*Kelch13^R539T^ parasites, RSA was carried out as described previously (46). In brief, schizonts (percoll-purified) were cultured until the early ring stage (0–3 h post-invasion) and subjected to varying concentrations of G-Rg1, DHA (700 nM), and their combination for 6 h. After incubation, parasites were washed and maintained for an additional 66 h. Parasitemia was determined by counting approximately 5,000 erythrocytes on Giemsa-stained smears. Additionally, to evaluate the effect of G-Rg1 on the survival of DHA-resistant parasites, early ring-stage (0–3 h) parasites were first treated with DHA (700 nM) for 6 h, followed by washing and subsequent exposure to G-Rg1 for 66 h. Parasite survival was assessed by scoring Giemsa-stained smears. Experiments were performed as three biological replicates, each *N* = 3.

### *In vivo* assay

The *in vivo* anti-malarial potential of G-Rg1, both as a monotherapy and in combination with ART, was evaluated in *P. berghei* ANKA-infected BALB/c mice (47). All experimental procedures were conducted in accordance with the guidelines established by the Institutional Animal Ethics Committee (IAEC) of Jawaharlal Nehru University (JNU, IAEC no. 38/2024). Mice obtained from Central Laboratory Animal Resources, JNU, were infected with 1 × 10^7^ parasites intraperitoneally at day 0 and segregated randomly into four groups (*n* = 5 mice/group). On day 2, mice in different groups were administered G-Rg1 (20 mg/kg in PBS, p.o.), ART (60 mg/kg in PBS, p.o.), G-Rg1 (10 mg/kg in PBS, p.o.) + ART (30 mg/kg in PBS, p.o.) and PBS (vehicle control), respectively, for 5 days. Parasitemia was quantified each day from Giemsa-stained tail blood smears. The mean survival time (in days) for mice in each experimental group post-inoculation was calculated over a 21-day observation period.

### Statistical analysis

Statistical analyses were done using GraphPad 8 (GraphPad Software Inc.), and *P* values were estimated by ANOVA and two-tailed Student’s *t*-test wherever required. Data are presented as means ± standard errors of means (SEM).

## Data Availability

Data will be made available on request.
